# Locked (Overlapping) Pubic Symphysis, an Uncommon Pelvic Ring Injury, Following High-Energy Crush Trauma: A Case Report

**DOI:** 10.7759/cureus.115895

**Published:** 2026-09-07

**Authors:** Murad Jwinate, Majdala Al-Bataineh, Ahmad Alawamleh, Mahmoud Sbeehat, Qusai Alazzam, Ashraf Albadaineh, Mohammad Al Dweeri, Mutaz Jaloody, Yazeed Zghoul, Laith Hseinat

**Affiliations:** 1 Department of Orthopaedics, Jordanian Royal Medical Services, Amman, JOR; 2 Faculty of Medicine, Yarmouk University, Irbed, JOR; 3 Department of Orthopaedics, Ministry of Health, Amman, JOR

**Keywords:** high-energy trauma, lateral compression, locked symphysis pubis, overlapping symphysis, pelvic ring, pelvis orif, polytrauma

## Abstract

Complex polytrauma resulting from high-energy trauma may necessitate interdisciplinary care. A rare pelvic injury called locked (overlapping) pubic symphysis is commonly linked to unstable pelvic ring disruption and frequently necessitates surgery.

A 25-year-old man sustained a high-energy crush injury after a large rock fell onto him. The patient presented to King Hussein Medical City Emergency Department on February 5, 2026. After initial assessment, imaging demonstrated a locked pubic symphysis with unstable pelvic ring disruption, a right sacral fracture extending into the neural foramina, lumbar compression fractures, left tibial and fibular shaft fractures, a left medial malleolus fracture, and retroperitoneal hematoma. Following initial resuscitation and stabilization, the patient underwent open reduction and internal fixation (ORIF) of the anterior pelvic ring symphyseal locked fracture dislocation with symphyseal plate, and the sacral fracture was treated conservatively, in addition to intramedullary nailing of the left tibial shaft fracture and ORIF to the medial malleolus with screws. The lumbar spine fractures were managed conservatively. He was discharged after 11 days of programmed rehabilitation and physical therapy without any neurological deficits or unexpected events.

This case highlights the importance of accurate imaging, meticulous preoperative planning, and multidisciplinary management in the treatment of locked pubic symphysis associated with complex polytrauma and the programmed rehabiliation. Early recognition, appropriate surgical intervention, and individualized conservative management of the posterior pelvic ring were associated with restoration of pelvic stability and a favorable clinical outcome in this case.

## Introduction

Polytrauma is still a major cause of death for young adults and is often linked to high-energy mechanisms of damage. These patients frequently suffer pelvic and long-bone fractures, which can cause a systemic inflammatory response, shock, and hemodynamic instability. The therapy of seriously injured patients still depends on damage-control principles, early bleeding control, physiologic stabilization, and prompt surgical intervention [1,2].

Pelvic fractures account for approximately 2%-3% of all fractures and are most caused by high-energy trauma such as motor vehicle collisions and falls from height. In polytrauma patients, unstable pelvic ring injuries are associated with substantial hemorrhage, vascular injury, and injuries involving the abdomen, spine, chest, and lower extremities, contributing to prolonged hospitalization, intensive care admission, and increased mortality [3-5].

Hemodynamically unstable pelvic ring fractures rank among the most serious injuries in trauma surgery because of the significant risk of extensive retroperitoneal bleeding. The fundamentals of initial care continue to be early physiologic evaluation, timely bleeding control, pelvic stability, and adequate imaging. To guide treatment planning, computed tomography (CT) and, where necessary, angiography are essential for identifying fracture patterns, related injuries, and sources of bleeding. Treatment planning is made easier by the precise evaluation of fracture morphology, fragment displacement, pelvic ring instability, and related visceral injuries made possible by multiplanar and three-dimensional CT reconstruction [6,7]. Depending on the strength and duration of the compressive force, high-energy crush injuries may further complicate musculoskeletal and soft-tissue injury [8]. A lateral compression mechanism is usually the cause of the uncommon pelvic injury known as locked pubic symphysis. Unlike routine pubic symphyseal diastasis, in which the pubic bones separate without interlocking, locked pubic symphysis occurs when one pubic bone displaces across the midline and becomes trapped behind the contralateral pubic bone, often within the contralateral obturator foramen. This mechanical interlocking may prevent successful closed reduction, particularly when the pubic bone is incarcerated, making open reduction necessary in irreducible cases. Urethral injuries are often associated with this injury pattern [9, 10]. In this report, we present a young male patient who sustained a rare locked pubic symphysis following high-energy crush trauma in the setting of complex polytrauma, including a sacral fracture, lumbar spine fractures, and ipsilateral lower-extremity fractures. The case is particularly noteworthy because, following anatomical reduction and anterior fixation, the posterior pelvic ring remained stable without requiring posterior fixation, with maintained alignment on postoperative imaging and subsequent follow-up. This case highlights the importance of detailed three-dimensional imaging, careful intraoperative assessment of pelvic ring stability, and individualized postoperative management in this uncommon injury pattern.

## Case presentation

On February 5, 2026, a 25-year-old male patient was admitted to the emergency department after a large rock collapsed onto him, causing a high-energy crush injury involving the pelvis and left lower extremity. He sustained multiple fractures involving the pelvis, lumbar spine, and left lower limb tibial shaft and medial malleolus. Upon presentation, the patient was tachycardic and hypotensive. Initial stabilization and resuscitation were performed according to Advanced Trauma Life Support (ATLS) protocols. A pelvic binder had been applied by the emergency medical services (EMS) because of suspected pelvic instability. On physical examination, severe perineal and scrotal ecchymosis, a prominent suprapubic contusion, marked tenderness over the anterior pelvic ring with a palpable bony step-off, and an obvious deformity of the left lower leg were noted. Given the risk of associated genitourinary injury, a formal urological consultation was obtained in the emergency department. There was no blood at the urethral meatus or hematuria, and no evidence of urethral or bladder injury. Urinary catheterization was performed by the trauma surgeon in the presence of the urologist without difficulty. Retrograde urethrography and cystography were not performed. Subsequent imaging and laboratory investigations were obtained to characterize the extent of injury.

Computed tomography (CT) and magnetic resonance imaging (MRI) demonstrated extensive injuries, including a comminuted fracture of the left superior pubic ramus, a displaced fracture of the left inferior pubic ramus, pubic symphysis diastasis with unstable pelvic ring disruption, and a displaced right sacral body fracture extending into the neural foramina. Based on the mechanism of injury, the pelvic ring injury was considered a lateral compression-type injury. However, the injury pattern did not fit the conventional Young-Burgess lateral compression classification, as the left superior pubic ramus fracture was associated with a contralateral right sacral fracture. The left superior pubic ramus fracture was comminuted, while the right sacral fracture was classified as a Denis zone II fracture. On CT, the right sacral fracture demonstrated less than 2 mm of displacement, while the pubic symphyseal displacement measured 14 mm. Spinal MRI showed acute compression fractures involving the superior endplates of L2, L3, and L4, accompanied by bone marrow edema and multiple left-sided transverse process fractures involving L2, L3, and L4. Left lower extremity evaluation showed comminuted fractures of the left tibial shaft and fibula, in addition to a left medial malleolus fracture.

Systemic trauma screening showed no evidence of acute intracranial hemorrhage or skull fractures on CT head imaging, apart from an incidental sclerotic lesion involving the right mandibular condyle. CT chest demonstrated bi-basal atelectatic changes without pneumothorax or pleural effusion. Abdominopelvic imaging revealed retroperitoneal and presacral hematomas secondary to pelvic ring disruption, with a small peri-splenic fluid collection and minimal fluid tracking within the right paracolic gutter; both were managed conservatively. CT angiography of the lower extremities demonstrated preserved bilateral arterial patency.

The initial CT assessment suggested a pubic symphysis diastasis with associated pelvic ring fractures. However, the presence of overlapping pubic bodies crossing the midline on three-dimensional CT reconstruction, rather than simple separation of the pubic bones, was recognized as a key radiographic feature of a locked pubic symphysis. During detailed preoperative planning, a careful review of the three-dimensional CT reconstruction demonstrated that the pubic bodies had overridden across the midline, resulting in a locked pubic symphysis rather than a simple symphyseal diastasis (Figure 1). This finding changed the planned reduction strategy.

**Figure 1:**
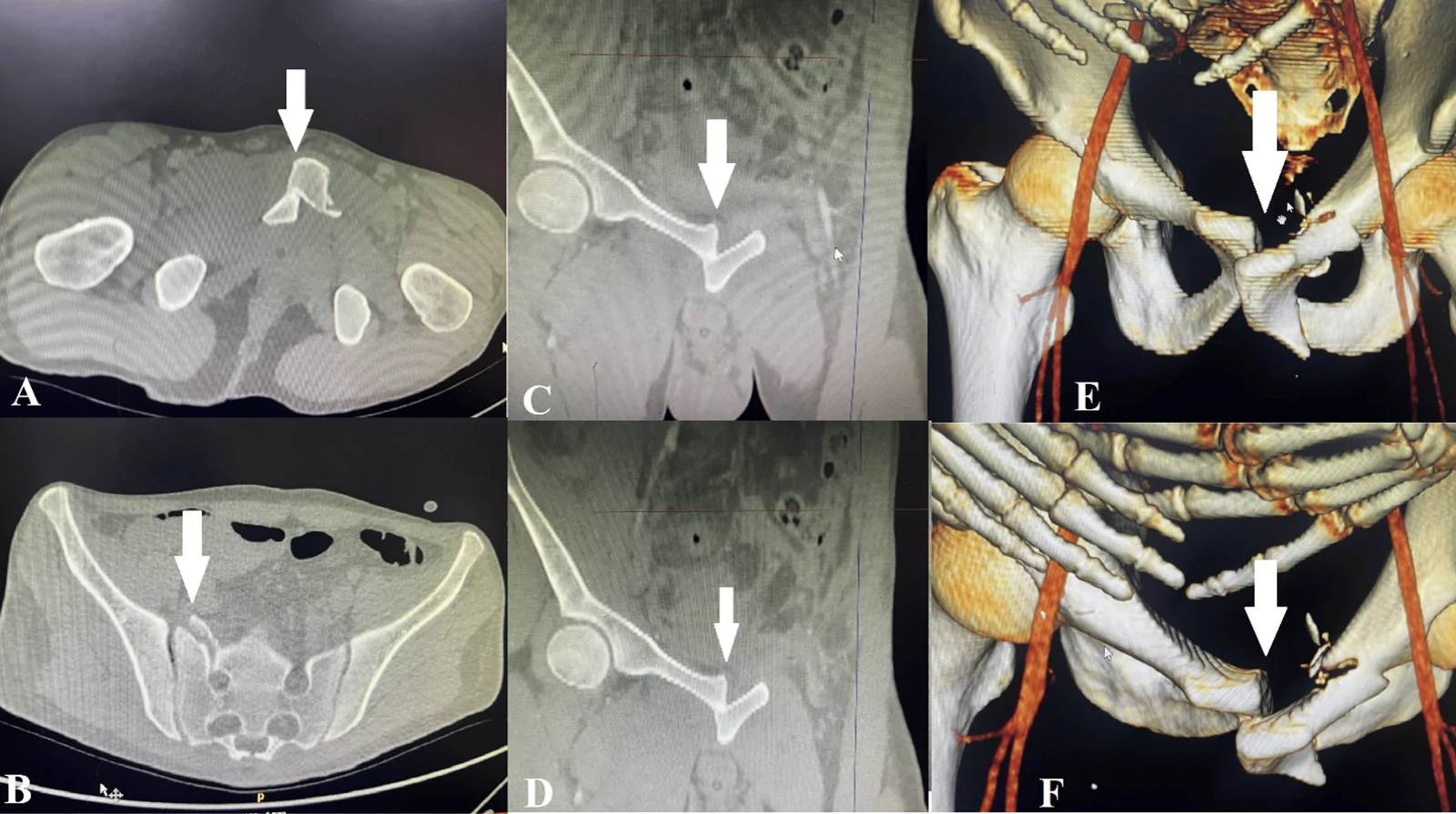
Initial CT Imaging Demonstrating Locked Pubic Symphysis and Associated Pelvic Injuries (A) Initial axial computed tomography (CT) image demonstrating anterior displacement with overlap and locking of the left pubic bone anterior to the right pubic bone (arrow). (B) Initial axial CT image demonstrating a displaced right sacral body fracture extending into the sacral neural foramina (arrow). (C, D) Initial coronal CT images demonstrating fracture-dislocation of the pubic symphysis with overlap of the pubic bones (arrow). (E, F) Initial three-dimensional (3D) CT reconstructions demonstrating anterior displacement with overlap and locking of the left pubic bone anterior to the right pubic bone (arrow).

The patient remained hospitalized for 11 days under the care of the trauma and orthopedic teams. During the first days of admission, the patient was hemodynamically stabilized, pain control was maintained, serial neurovascular examinations were performed, and the pelvic binder was removed while the left lower extremity was temporarily splinted until definitive fixation.

Surgical fixation was carried out on February 9, 2026. Through a Pfannenstiel approach, open reduction and internal fixation (ORIF) of the pubic symphysis and pelvic ring fractures were performed. Intraoperatively, the locked pubic symphysis was confirmed, and reduction was achieved using a controlled distraction technique with cortical screws inserted into the superior pubic rami and a lamina spreader to disengage the locked pubic bodies before definitive fixation with a pelvic reconstruction plate (Figure 2). A six-hole Synthes reconstruction plate was used for superior fixation across the pubic symphysis and the left superior pubic bone. Five 3.5-mm cortical screws were inserted, including three screws over the right symphyseal crest and two screws in the right pubic bone. Following reduction and fixation of the anterior pelvic ring, the posterior pelvic ring was reassessed intraoperatively and was found to be stable, with no further displacement following manipulation and reduction of the anterior ring. Given the prolonged operative course and the anesthesiologist’s recommendation to limit further operative time, posterior fixation was not performed, with the option of reassessment and delayed fixation if subsequently indicated. In addition, intramedullary (IM) nailing of the left tibial shaft fracture and the ORIF to the medial malleolus was performed to achieve fracture stabilization, restore alignment, and facilitate early mobilization.

**Figure 2:**
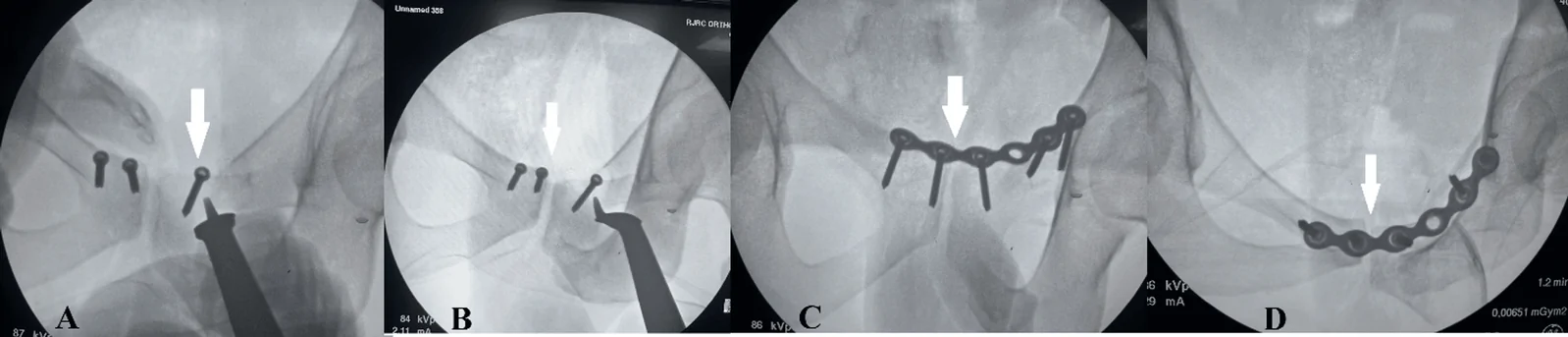
Intraoperative Reduction and Definitive Fixation of the Locked Pubic Symphysis (A, B) Intraoperative fluoroscopic images demonstrating the push-pull reduction technique, in which cortical screws and a lamina spreader were used to reduce the overlapping left pubic bone to its anatomical position and restore the pubic symphysis. (C, D) Final intraoperative fluoroscopic pelvic outlet (C) and pelvic inlet (D) views demonstrating anatomical reduction of the pubic symphysis and definitive fixation using reconstruction plates for both the pubic symphysis and the left superior pubic ramus fracture, with satisfactory implant positioning.

The lumbar spine injuries were treated conservatively after MRI demonstrated no substantial spinal canal compression, spinal instability, or disruption of the posterior ligamentous complex.

Immediate postoperative anteroposterior pelvic radiographs demonstrated satisfactory anatomical reduction of the pubic symphysis, restoration of the pelvic ring alignment, and stable fixation of both the pubic symphysis and the left superior pubic ramus fracture. Postoperative computed tomography (CT) was performed to reassess the posterior pelvic ring and further confirmed anatomical reduction of the previously locked pubic symphysis, appropriate reduction of the left superior pubic ramus fracture, satisfactory positioning of the reconstruction plates and screws, and maintained posterior pelvic ring alignment without further displacement (Figure 3). Based on the intraoperative assessment and postoperative CT findings, conservative management of the posterior pelvic ring injury was continued.

**Figure 3:**
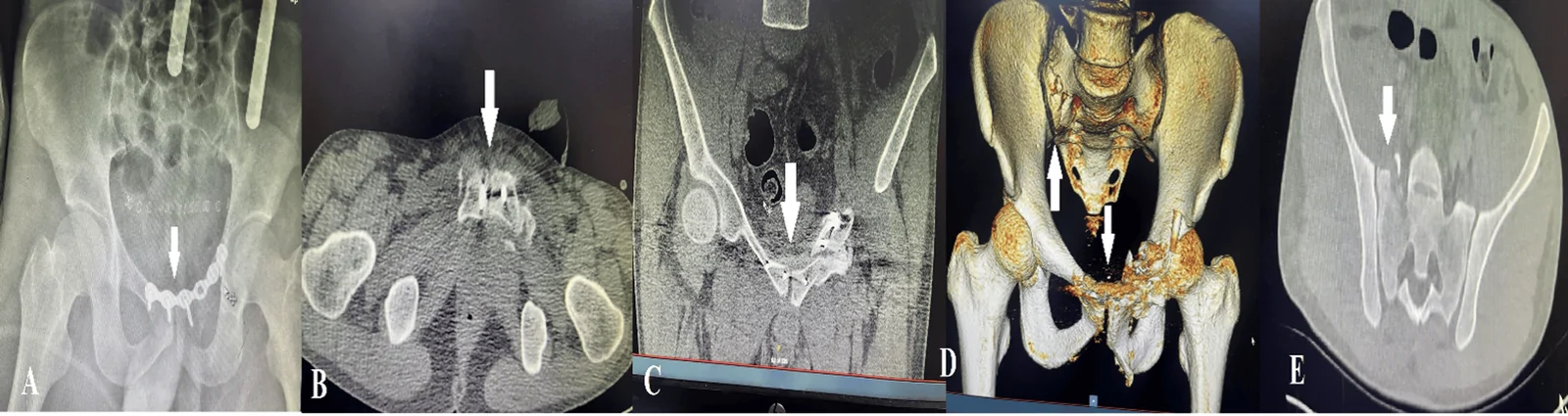
Postoperative Imaging Demonstrating Restoration of Pelvic Ring Alignment (A) Immediate postoperative anteroposterior pelvic radiograph demonstrating anatomical reduction of the pubic symphysis, satisfactory positioning of the reconstruction plate (arrow), and restoration of the right sacroiliac joint alignment. (B, C) Immediate postoperative axial (B) and coronal (C) computed tomography (CT) images confirming anatomical reduction of the pubic symphysis and appropriate reduction of the left superior pubic ramus fracture. (D) Three-dimensional (3D) CT reconstruction demonstrating restoration of the pelvic ring anatomy and satisfactory implant positioning. (E) Axial CT image confirming anatomical reduction of the right sacroiliac joint without residual displacement.

Postoperatively, the patient followed a structured multidisciplinary rehabilitation protocol. Because of the associated left ankle fracture, weight-bearing was initially restricted. During the first two weeks, rehabilitation focused on bedside mobilization, including sitting, regular position changes, active-assisted range-of-motion exercises of the hips, knees, and ankles, and supervised transfers from the bed to a chair.

At two weeks postoperatively, the surgical wounds had healed uneventfully, and the patient commenced progressive partial weight-bearing with the assistance of a walker under physiotherapy supervision. At six weeks postoperatively, clinical and radiographic follow-up demonstrated maintained fracture reduction, stable implant position, and no further posterior pelvic displacement on follow-up radiographs and CT. The patient reported no pain during ambulation and demonstrated satisfactory ambulatory status. Based on the clinical assessment and maintained radiological alignment, progression to full weight-bearing was permitted. At three months following surgery, a formal strengthening program targeting the hip, pelvic girdle, and lower extremity musculature was initiated to restore functional capacity and facilitate a gradual return to normal daily activities.

At the six-month follow-up, the patient demonstrated maintained anatomical reduction of the pubic symphysis and right sacroiliac joint, complete union of the left superior pubic ramus, left tibial shaft, and medial malleolar fractures, with no evidence of implant loosening, loss of reduction, or hardware failure (Figure 4). Clinically, the patient had returned to full weight-bearing without assistance and had a painless functional range of motion of the left hip, knee, and ankle. No residual pelvic instability, mechanical pelvic pain, gait abnormality, or neurological deficit was observed, and the patient had resumed normal daily activities without functional limitation or disability.

**Figure 4:**
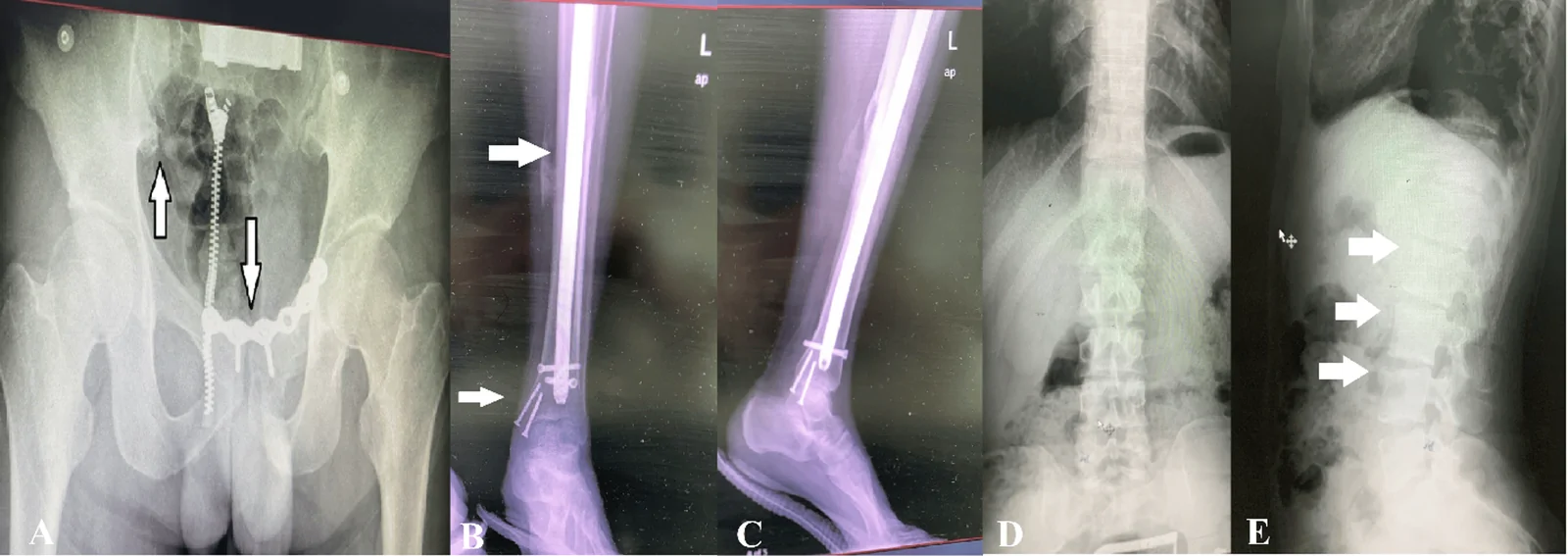
Six-Month Follow-Up Imaging Demonstrating Fracture Union and Maintained Alignment (A) Six-month follow-up anteroposterior pelvic radiograph demonstrating maintained anatomical reduction of the pubic symphysis and right sacroiliac joint (arrow), complete union of the left superior pubic ramus fracture, and stable implant positioning (arrow). (B,C) Six-month follow-up anteroposterior and lateral radiographs of the left tibia (arrow) and ankle (arrow) demonstrating complete fracture union with maintained alignment. (D, E) Six-month follow-up anteroposterior (D) and lateral (E) radiographs of the lumbar spine demonstrating maintained alignment of the L2, L3, and L4 vertebral fractures without further vertebral collapse or progression of deformity following conservative management (arrow).

## Discussion

High-energy trauma remains a common cause of polytrauma in young adults. These patients often have long-bone and pelvic fractures, which may be followed by shock or hemodynamic instability. Trauma frequently affects several body parts in individuals with serious injuries, including the chest, abdomen, pelvis, spine, and extremities. The patient's physiological state affects the course of treatment, especially if resuscitation and interim stabilization are necessary prior to final fracture fixation. Imaging tests are crucial for the assessment of related spinal injuries because a neurological examination may not reveal major fractures. While magnetic resonance imaging (MRI) offers additional assessment of spinal cord and soft-tissue injuries and helps inform later care decisions, CT continues to be the primary imaging modality for the initial assessment of spinal trauma. While therapy is mostly dictated by the existence of neurological deficits, spinal cord compression, and mechanical instability, stable fractures without significant neurological damage may be treated conservatively [11-13].

Unstable pelvic ring injuries are among the most severe consequences of high-energy trauma and are frequently associated with substantial blood loss and hemodynamic compromise. Arterial veins, venous plexuses, or exposed cancellous bone surfaces can all cause bleeding, and retroperitoneal hemorrhage can spread from the pelvis to the paravertebral area, flanks, gluteal area, thighs, and abdominal retroperitoneal regions. Prior to final fracture fixation, early therapy prioritizes restoration of hemodynamic stability, bleeding control, and physiologic resuscitation. Shorter stays in the critical care unit, reduced morbidity, and better clinical results have all been linked to early stabilization. CT remains an essential imaging modality for identifying fracture patterns and determining the presence and extent of pelvic hemorrhage [14-17].

Sacral fractures and posterior pelvic ring injuries after high-energy trauma are commonly linked to pelvic ring injuries. Retroperitoneal bleeding and potentially fatal instability may arise from disruption of the posterior ligamentous complex and the pubic symphysis, which play a major role in the stability of the pelvic ring. In order to reduce discomfort, neurological issues, irregularities in gait, and long-term functional impairment, restoring posterior pelvic stability continues to be the main objective of treatment [16-19].

Damage Control Orthopedics (DCO) was developed to reduce the physiological burden of prolonged surgical procedures in severely injured patients. By prioritizing hemorrhage control and temporary fracture stabilization before definitive fixation, DCO minimizes the “second-hit” inflammatory response and has become an accepted strategy in the management of unstable pelvic fractures [20].

Locked (overlapping) pubic symphysis has been classified according to the ability to achieve and maintain reduction, the presence of incarceration within the contralateral obturator foramen, and the severity of associated posterior pelvic ring injuries. Grade I injuries can usually be managed with closed reduction, whereas Grade II and Grade III injuries generally require open reduction. Persistent pelvic instability following reduction necessitates anterior fixation, with posterior fixation performed when significant posterior ring injuries are present. Several reduction techniques have been described, including closed maneuvers using the femur as a lever arm, longitudinal traction, direct pressure over the pubic symphysis, and lateral pelvic compression or distraction and push-pull technique. However, mechanical entrapment and associated injuries may prevent successful closed reduction, making open reduction the preferred treatment in irreducible cases [10,21].

An efficient minimally invasive method for stabilizing the posterior pelvic ring is percutaneous sacroiliac screw fixation. However, because fluoroscopy may underestimate fracture displacement and screw malposition may induce neurovascular, urological, or gastrointestinal problems, precise screw placement is technically challenging. Intraoperative visibility is enhanced and more precise screw insertion is made possible by three-dimensional CT-based navigation [22].

Urethral injury is the most common associated injury in patients with locked pubic symphysis. It results from the crush and shearing forces generated during displacement of the pubic bone. Therefore, careful urological evaluation, including urethrography when indicated, is recommended because urethral stricture remains the most frequent long-term complication [23].

High-energy trauma can cause complicated injury patterns, such as locked pubic symphysis, unstable pelvic ring disruption, sacral fractures, spinal fractures, and lower-extremity fractures, as our case illustrates. The need for timely physiologic stabilization and cautious multidisciplinary therapy was further highlighted by the existence of retroperitoneal hematoma. For precise characterization of the damage pattern and suitable surgical planning, a comprehensive imaging examination was necessary. According to the literature that is currently accessible, early resuscitation followed by final fixation had a positive outcome without neurological impairment. In our case, detailed three-dimensional CT reconstruction was essential for recognizing the locked pubic symphysis and guiding the surgical strategy. Following anterior reduction and fixation, the posterior pelvic ring remained stable without further displacement, allowing conservative management without posterior fixation. The patient’s weight-bearing progression was guided primarily by the associated ankle fracture initially, followed by clinical and radiographic assessment, with full weight-bearing achieved at six weeks.

## Conclusions

This case demonstrates the complex injury pattern that can arise from high-energy crush trauma, including lower-extremity fractures, sacral fractures extending into the neural foramina, locked pubic symphysis, and lumbar spine fractures. Appropriate management in this case required precise radiological evaluation, detailed preoperative planning, careful assessment of pelvic ring stability, and multidisciplinary management. The favorable outcome in this patient highlights the importance of recognizing locked pubic symphysis on detailed CT imaging, achieving appropriate reduction and stabilization of the anterior pelvic ring, and individualized management of the posterior pelvic ring and associated injuries.
